# 应用基因表达谱芯片从抗脱落凋亡肺癌A549细胞中筛选转移相关基因

**DOI:** 10.3779/j.issn.1009-3419.2010.01.04

**Published:** 2010-01-20

**Authors:** 凯 苏, 杰 雷, 伟 张, 志培 张, 小飞 李, 勇安 周, 平 张, 小平 王

**Affiliations:** 1 710038 西安，第四军医大学唐都医院胸腔外科 Department of Toracic Surgery, Tangdu Hospital, the Fourth Military Medical University, Xi'an 710038, China; 2 710032 西安，第四军医大学基础部生物技术中心 Center for Genetic Engineering and Biotechnology, the Fourth Military Medical University, Xi'an 710032, China

**Keywords:** 肺肿瘤, 抗脱落凋亡, 肿瘤转移, 人类基因组微阵列, 基因表达谱, Lung neoplasms, Anoikis resistant, Tumor metastasis, Human Genome Array, Gene expression profle

## Abstract

**背景与目的:**

正常上皮或内皮细胞脱离细胞外基质会发生脱落凋亡，但肿瘤细胞可不依赖细胞基质生长而不发生凋亡，这一现象被称为抗脱落凋亡，目前国内外相关研究均认为抗脱落凋亡是肿瘤发生转移的始动环节。本实验旨在比较抗脱落凋亡及正常贴壁生长肺腺癌A549细胞基因组表达差异，从中筛选肺癌转移相关基因。

**方法:**

利用多聚羟乙基甲基丙烯酸树脂处理培养皿，致细胞无法贴壁生长，建立抗脱落凋亡肺癌A549细胞系；利用北京博奥生物芯片公司的人类V2.0全基因组寡核苷酸微阵列芯片，检测其与正常贴壁生长A549细胞的基因表达差异性，筛选肺癌转移相关基因。

**结果:**

共得到表达差异的基因745个，从中筛选出63个与肺癌转移密切相关的基因。

**结论:**

成功建立抗脱落凋亡肺癌A549细胞系并筛选出转移相关差异表达基因，为进一步研究肺癌转移相关信号转导通路及其它相关研究提供依据。

## Abstract (en)

**Background and objective:**

As a barrier to metastases, cells normally undergo apoptosis after they lose contact with their extra cellular matrix (ECM). This process has been termed "anoikis". Tumour cells that acquire malignant potential have developed mechanisms to resist anoikis and thereby survive after detachment from their primary site while traveling through the lymphatic and circulatory systems. This "anoikis resistance" is considered the first step to tumor metastases. The aim of this study was to screen metastasis-associated genes from anoikis resistant and adherent growth A549 lung cancer cell by Human Genome Array.

**Methods:**

Establish anoikis resistant A549 lung cancer cell lines by using poly-hydroxyethyl methacrylate resin processed petri dishes, which causes cell free from adherent. The different expressed gene between anoikis resistant A549 cell and adherent growth A549 cell was tested using human V2.0 whole-genome oligonucleotide microarray, a product of Capitalbio Corporation, Beijing. Screen metastasis-associated genes.

**Results:**

745 different expressed genes were screened, including 63 highly metastasis-associated genes.

**Conclusion:**

The successfully established anoikis resistant A549 cell lines and screened different expressed genes provide us basis for further research on metastasis of lung cancer.

细胞与基质之间的相互作用对于细胞的某些表型具有重要意义，如果失去基质的支持，如某些不具备转移特性的实体瘤细胞和正常上皮细胞从原位脱落进入血流，或在体外培养的情况下人为阻断细胞的粘附，细胞将发生程序性死亡，这种现象被称为“anoikis”^[1]^，即脱落凋亡或失巢凋亡。肿瘤细胞一旦开始发生转移，即获得了抗脱落凋亡的能力。这种存在于某些肿瘤细胞的抗脱落凋亡，是转移瘤发生的重要原因之一^[2]^。目前，关于脱落凋亡/抗脱落凋亡的研究仍局限在个别基因、个别通路、抗脱落凋亡机理的认识仍不系统，细胞的多样性以及肿瘤细胞的易突变也赋予不同的肿瘤细胞不同的抗脱落凋亡机制。我们应用高侵袭性肺腺癌A549细胞系，分别在贴壁与悬浮两种状态下培养，检测两种不同生长状态肺癌A549细胞系基因表达上的差异，旨在高通量地筛选肺癌抗脱落凋亡相关基因，并从中筛选与肺癌转移密切相关的基因，为进一步研究肺癌转移侵袭性提供数据。

## 1 材料和方法

### 1.1 实验材料

RPMI-1640培养基购自Gibco公司。胎牛血清（FBS）购自杭州四季青生物制品公司。多聚羟乙基甲基丙烯酸树脂（Poly-HEMA）购自Sigma公司。DNA提取试剂盒为TIANamp血液/细胞/组织基因组DNA提取试剂盒。细胞系：高侵袭性肺腺癌A549细胞系由第四军医大学基础部生化教研室王江博士惠赠。细胞培养于含有10%FBS的RPMI-1640培养液中，置37 ℃、5%CO_2_孵箱中培养。

### 1.2 实验方法

#### 1.2.1 细胞悬浮培养皿的准备及细胞悬浮培养^[3, 5]^

Poly-HEMA溶于无水乙醇（10 g/L）。每个55 mm直径的petri培养皿加2 mL Poly-HEMA溶液，室温下待乙醇完全挥发后，再加1遍Poly-HEMA溶液。临用前，petri培养皿以PBS洗4遍，置于超净台中紫外线照射消毒待用。以胰酶消化贴壁的肺癌细胞，收集细胞，计数并接种于poly-HEMA处理过的petri培养皿中。每个培养皿加入1×10^6^个细胞，置37 ℃、5%CO_2_孵箱中培养72 h，每天观察细胞状态并计数。由于Poly-HEMA不带电荷，细胞无法贴壁，只能在培养皿中脱落生长，借此来模拟细胞的脱落培养状态，对照组细胞为贴壁培养的细胞。

#### 1.2.2 细胞质梯度DNA测定

收集悬浮及贴壁细胞，各取1×10^6^个。紫外灯下观察DNA ladder ^[3, 4]^。

#### 1.2.3 流式细胞术检测

收集悬浮及贴壁细胞，各取1×10^6^个细胞转移至离心管，然后用50 μL结合缓冲液重悬细胞，每组分别加入PI及AnnexinV2FITC，室温避光30 min上样。立即用流式细胞仪分析凋亡率。

#### 1.2.4 芯片制备

以贴壁培养A549细胞为对照组，抗脱落凋亡A549细胞为实验组，利用北京博奥生物芯片公司的人类V2.0全基因组寡核苷酸微阵列芯片（35K Human Genome Array），检测两种细胞的基因表达差异。

#### 1.2.5 数据检索

利用北京博奥生物芯片公司CB-MAS 4.0生物分子功能注释系统筛选表达差异基因，利用NCBI Pubmed数据库进一步筛选与肺癌侵袭转移密切相关的基因。

## 2 结果

### 2.1 抗脱落凋亡肺癌A549细胞培养

在细胞悬浮培养过程中我们观察到，A549肺癌细胞脱落培养12 h后，细胞开始聚集，24 h后细胞相互聚集成比较大的细胞团块，且培养时间越长，聚集成的细胞团块越大、越致密。但较贴壁正常生长A549细胞相比，悬浮培养细胞生长速度明显变缓，细胞形态差异明显（图 1）。

**1:**
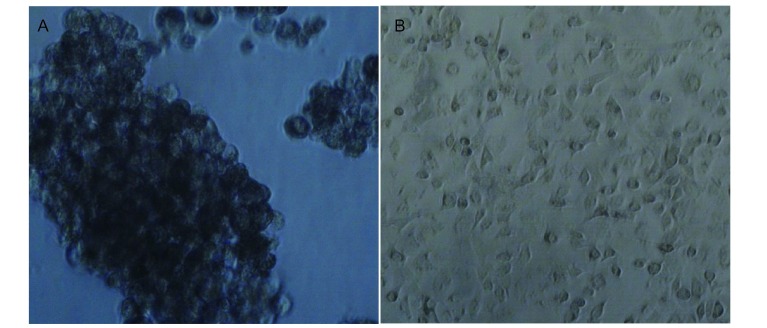
细胞形态学表现（×100） Cell morphology (×100)

### 2.2 抗脱落凋亡肺癌A549细胞检测

收集悬浮及正常贴壁生长72 h细胞进行细胞质梯度DNA分析，结果显示两种状态培养的肺腺癌A549细胞电泳图像均未出现凋亡特异性梯度条带（图 2），说明细胞未发生凋亡。流式细胞检测结果与细胞质梯度DNA测定结果一致（图 3），两种培养状态下肺癌细胞培养72 h未检测到细胞明显凋亡（细胞凋亡发生率均小于5%）。

**2:**
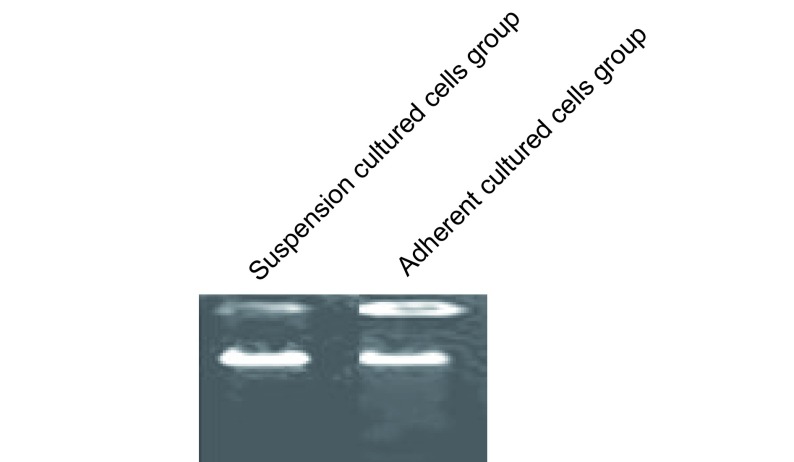
DNA电泳 DNA electrophoresis

**3:**
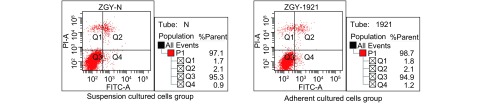
各组流式细胞凋亡率 The percent of apoptotic cells in different groups by FCM

### 2.3 人基因表达谱芯片检测到的表达差异基因

基因芯片检测（图 4）共得到表达差异的基因745个（Ratio≥2.0或Ratio≤0.5），其中实验组相对对照组上调2倍以上的基因数目有556个，实验组相对对照组下调0.5倍以上的基因数目有189个。所获得的基因通过NCBI Pubmed检索其功能。这些表达差异基因涉及原癌基因、抑癌基因、细胞周期蛋白、凋亡、免疫相关、信号转导、蛋白翻译合成及一些功能未知的基因。进一步筛选出63个与癌症转移密切相关的基因，其中上调基因42个，下调基因21个，在上调及下调基因中，部分基因经文献检索功能与本实验上下调关系不符，如上调基因为抑癌基因，下调基因为癌基因等，遂予以剔除，最终选定与本实验目的相关的介导肺癌转移的基因38个，其中上调基因25个，下调基因13个，列于下表（表 1，表 2）。

**4:**
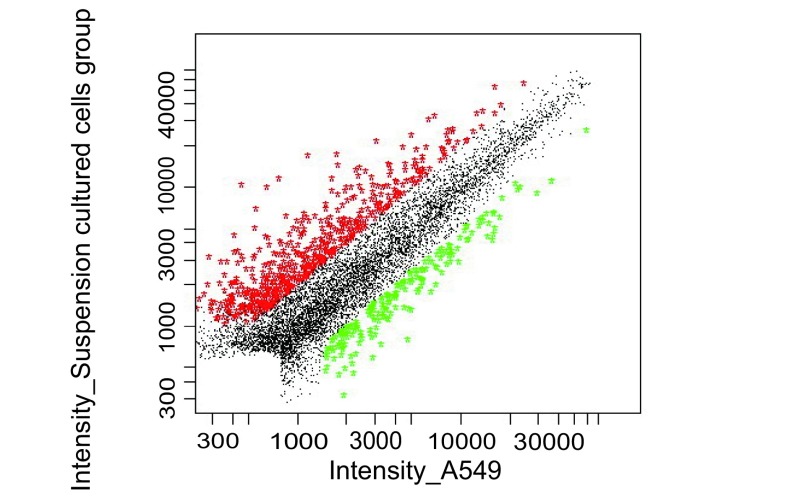
基因芯片散点图 Scattered plot graph of Cy3-labeled and Cy5-labeled probes hybirdizing with microarray

**1:** 上调基因及其功能简述 The up-regulated genes and brief introdutions of their functions

Name	Gene-id	Ratio	Function
*MET*	ENSG00000105976	9.991 8	The hepatocyte growth factor receptor, play a critical role in aggressive cancers such as breast cancer or SCLC.
*UGDH*	ENSG00000109814	9.941 3	The protein encoded by this gene plays roles in signal transduction, cell migration, and cancer growth and metastasis.
*C8orf4*	ENSG00000176907	8.241 3	This gene encodes protein that functions as a positive regulator of the Wnt/beta-catenin signaling pathway, which upstream regulator of the Wnt/beta-catenin pathway that enhances aggressive behavior of cancers.
*FOS*	ENSG00000170345	7.980 4	The FOS proteins have been implicated as regulators of cell proliferation, differentiation, and transformation. In some cases, expression of the FOS gene has also been associated with apoptotic cell death.
*SDCBP*	ENSG00000137575	5.800 5	The protein encoded by this gene enhanced tumor cell invasion and metastatic spread.
*ADAM10*	ENSG00000137845	5.212 3	Members of the ADAM family, interfere with cell-cell adhesion and enhance migration of cancer cells.
*ITGB1*	ENSG00000150093	4.883 7	Integrin family members are membrane receptors involved in cell adhesion and recognition in a variety of processes including embryogenesis, hemostasis, tissue repair, immune response and metastatic diffusion of tumor cells.
*MCL1*	ENSG00000143384	4.717 8	The protein encoded by this gene belongs to the Bcl-2 family. Te longer gene product enhances cell survival by inhibiting apoptosis while the alternatively spliced shorter gene product promotes apoptosis and is death-inducing.
*LAMP2*	ENSG00000005893	4.503 3	The protein encoded by this gene is a member of a family of membrane glycoproteins. It may play a role in tumor cell metastasis.
*CASP8*	ENSG00000064012	4.374 0	This gene encodes a member of the cysteine-aspartic acid protease (caspase) family, it plays a central role in the execution-phase of cell apoptosis. Coding polymorphisms of it may play a role in predisposition to lung cancer.
*ZNF217*	ENSG00000171940	4.311 7	The protein encoded by this gene might play a crucial role in the proliferation and invasion of ovarian cancer.
*HSPAS*	ENSG00000044574	4.300 9	Upregulation of this gene can signifcantly confer the chemoresistance to VP-16 in human lung cancer cell line SK-MES-1.
*CEACAM5*	ENSG00000105388	4.157 5	The protein encoded by this gene is a risk factor for brain metastasis development and is associated with poor prognosis in patients with advanced NSCLC.
*DUSP1*	ENSG00000120129	4.066 9	The protein encoded by this gene plays a role in progression of non-small cell lung cancer
*Q6P709_HUMAN*	ENSG00000143549	3.925 6	This gene encodes a member of the tropomyosin family of actin-binding proteins and it may play a role in cancer cell invasion and metastasis.
*HNRPH2*	ENSG00000126945	3.916 8	The hnRNPs are RNA binding proteins and they complex with heterogeneous nuclear RNA (hnRNA), and it plays a role in tumor cell proliferation.
*CCL20*	ENSG00000115009	3.890 3	Contribute to tumor growth and metastases.
*CAPG*	ENSG00000042493	3.890 0	This gene encodes a member of the gelsolin/villin family of actin-regulatory proteins that promotes cell invasion.
*PLAUR*	ENSG00000011422	3.872 7	A useful predictors of distant metastases, may interact with multiple integrins in normal human lung fbroblasts thereby promoting atachment, spreading, and migration.
*AXL*	ENSG00000167601	3.813 7	Activation of this gene may be a protective mechanism against hypertonicity-induced apoptosis, and is associated with lung adenocarcinoma and with tumor progression.
*EREG*	ENSG00000124882	3.695 3	A member of the epidermal growth factor family. Expression of this gene correlates with advanced disease, is EGFR dependent, and confers invasive properties on non-small cell lung cancer cells.
*CEACAM6*	ENSG00000086548	3.539 3	This gene silences reversed the acquired anoikis resistance of tumor cell lines and inhibited *in vivo* metastatic ability.
*PTGES*	ENSG00000148344	3.535 9	The protein encoded by this gene is associated with metastasis in non-small cell lung cancer.
*BNIP3*	ENSG00000197358	3.242	A survival mechanism during hypoxia-induced autophagy that promotes tumor progression.
*RAD21*	ENSG00000164754	2.984 8	This gene was closely related to the invasion and metastasis of cancer cells.

**2:** 下调基因及其功能简述 The down-regulated genes and brief introdutions of theri functions

Name	Gene-id	Ratio	Function
*MCM2*	ENSG00000073111	0.306 0	The protein encoded by this gene is an highly conserved mini-chromosome maintenance proteins. Expression of it was associated with the tumors' histological grade, existence of nodular metastases, malignancy on adenoma, and vascular invasion.
*CCND3*	ENSG00000112576	0.307 2	This protein encoded by this gene has been shown to interact with and be involved in the phosphorylation of tumor suppressor protein Rb.
*OSGIN1*	ENSG00000140961	0.318 6	A novel p53 target gene that is regulated by Peptidylarginine deiminase 4 and plays a role in apoptosis.
*FGFR1*	ENSG00000077782	0.320 7	A member of FGFR family, plays roles in cancer growth and metastasis.
*SLC3A2*	ENSG00000168003	0.323 2	This gene is a member of the solute carrier family and encodes a cell surface, transmembrane protein. It associates with integrins and mediates integrin-dependent signaling related to normal cell growth and tumorigenesis.
*HSPA1A*	ENSGOOOOO204389	0.373 4	This intronless gene encodes a 70 kDa heat shock protein,which induces proteasome-dependent degradationof HSP90 client proteins, G1 cell-cycle arrest, and extensive tumor-specifc apoptosis in human tumor cell lines.
*SFN*	ENSG00000175793	0.387 5	A lymph node metastasis-related protein in human lung squamous carcinoma and also enhance p53 degradation, thereby inhibiting p53-mediated cell death.
*TACC3*	ENSG00000013810	0.399 8	The function of this gene is that it may be involved in cell growth and differentiation. Expression of this gene is up-regulated in some cancer cell lines.
*EVL*	ENSG00000196405	0.421 4	This gene is suppressed in human cancers such as colorectal.
*PA2G4*	ENSG00000170515	0.426 7	This gene encodes an RNA-binding protein that is involved in growth regulation. It suppresses gene transcription and tumorigenesis of prostate cancer cells.
*E2F1*	ENSG00000101412	0.443 4	The protein encoded by this gene is a member of the E2F family of transcription factors. It can mediate both cell proliferation and p53-dependent/independent apoptosis.
*DDX18*	ENSG00000088205	0.473 1	This gene is important for cell proliferation and that its inhibition could prevent tumor cell proliferation
*Q5SQ38_HUMAN*	ENSGOOOOO204463	0.485 2	This gene encodes a nuclear protein that is implicated in the control of apoptosis. It forms a complex with E1A binding protein p300 and is required for the acetylation of p53 in response to DNA damage.

## 3 讨论

侵袭和转移是造成各种恶性肿瘤患者死亡的主要原因，且是一个极其复杂、涉及肿瘤与宿主间相互作用的多因素过程，包括瘤细胞从原发肿瘤脱落，进入细胞外基质与脉管内，直至在远端适宜组织中克隆生长，期间受到许多相关基因的调控。有学者通过对肺癌、肠癌、卵巢癌、恶性黑色素瘤、口腔鳞状细胞癌等恶性肿瘤细胞株体外悬浮培养，并通过DNA ladder和流式细胞仪进行与相应正常组织细胞相比，检测均发现恶性肿瘤细胞的抗失巢凋亡能力明显增强；同时，具有高转移能力的细胞株的抗脱落凋亡能力明显强于低转移细胞株；另外，在裸鼠体内进行的肿瘤细胞侵袭实验也证实，高抗脱落凋亡细胞株转染的裸鼠全身脏器转移率高于对照组^[5-10]^。这一系列试验验证了抗脱落凋亡是肿瘤侵袭转移的始动环节，并提示此环节是受到相关基因的严密调控。但是目前抗脱落凋亡的相关基因及其对肿瘤细胞侵袭、转移作用的影响并不十分清楚。因此较全面地探明肿瘤抗脱落凋亡相关基因及其对细胞侵袭、转移功能的影响具有重要的科研和临床价值。

鉴于肿瘤细胞的多样性及易突变性，不同的肿瘤细胞可能存在着不同的抗失巢凋亡机制，我们选择高侵袭性肺腺癌A549细胞系作为研究对象，首先尝试构建抗脱落凋亡的A549细胞系。本实验证明，肺癌A549细胞可以悬浮生长不发生凋亡，但出现成团块样生长。悬浮培养的A549细胞成团块样生长，我们猜测可能部分代替了细胞与基质间互相作用，使肿瘤细胞可在非生理环境下存活。然而，细胞悬浮状态下生长缓慢，说明团块样生长并不能完全代替细胞与基质间全部生理功能，仅能维持肿瘤细胞在不利的环境下生存的最低需要。A549细胞的成功悬浮培养，在体外模拟了高侵袭性肿瘤细胞自原发部位侵入循环系统远处转移过程中的生存状态，为体外实验研究恶性肿瘤转移机制提供了很好的模型。

肿瘤的发生发展是一个涉及细胞多基因的作用过程，目前的研究大都仅仅涉及个别基因，无法从全局的角度宏观地认识肺癌的进展。基因芯片则具有高效、灵敏、高通量地分析细胞内基因表达谱的优势。我们的研究筛选了745个表达差异基因，并将基因芯片检查筛选出的表达差异的基因通过NCBI Pubmed进行功能比对。结果显示：这些表达差异基因涉及原癌基因、抑癌基因、细胞周期蛋白、凋亡、免疫相关、信号转导、蛋白翻译合成及一些功能未知的基因，表明肿瘤侵袭转移是一个涉及多种基因、多类信号转导通路通路并涉及多个细胞事件的复杂过程^[11, 12]^。我们从中筛选肺癌侵袭转移始动环节密切相关的63个基因，但某些筛选出的基因已知功能与上调、下调比例不符，如部分抑癌基因等预期应表达下调的基因在试验中表达为上调，而部分癌基因表达则为下调，虽然有某些侵袭性肿瘤确实存在某些基因会因肿瘤类型不同而部分上调，部分下调的情况，但我们目前的实验仍不能排除假阳性，这一部分数据我们正在进一步研究。而最终筛选出的38个与肺癌转移密切相关的基因，为今后系统的研究肺癌转移的相关机制打下了坚实基础。
